# Intraepithelial Lymphogram in the Diagnosis of Celiac Disease in Adult Patients: A Validation Cohort

**DOI:** 10.3390/nu16081117

**Published:** 2024-04-10

**Authors:** Carlota García-Hoz, Laura Crespo, Roberto Pariente, Ana De Andrés, Rafael Rodríguez-Ramos, Garbiñe Roy

**Affiliations:** 1 Department of Immunology, University Hospital Ramón y Cajal, Instituto Ramón y Cajal de Investigación Sanitaria, 28034 Madrid, Spain; roberto.pariente@salud.madrid.org (R.P.); aandresm@salud.madrid.org (A.D.A.); rafael.rodriguez.ramos@salud.madrid.org (R.R.-R.); groy.hrc@salud.madrid.org (G.R.); 2 Department of Gastroenterology, University Hospital Ramón y Cajal, Instituto Ramón y Cajal de Investigación Sanitaria, 28034 Madrid, Spain; lcreper@yahoo.es

**Keywords:** celiac disease (CD), gluten, duodenal intraepithelial lymphocytes (IEL), lymphogram

## Abstract

Background: Celiac disease is a gluten-related pathology, highly prevalent and heterogeneous in its clinical presentation, which leads to delays in diagnosis and misdiagnosis. The analysis of duodenal intraepithelial lymphocytes (IELs) by flow cytometry (lymphogram) is emerging as a discriminative tool in the diagnosis of various forms of celiac disease (CD). Aims: The aim of this study was to validate IEL lymphogram performance in the largest adult series to our knowledge, in support of its use as a diagnostic tool and as a biomarker of the dynamic celiac process. Methods: This was a retrospective study including 768 adult patients (217 with active CD, 195 on a gluten-free diet, 15 potential CD patients, and 411 non-celiac controls). The IEL subset cut-off values were established to calculate the diagnostic accuracy of the lymphogram. Results: A complete celiac lymphogram profile (≥14% increase in T cell receptor [TCR]γδ IELs and simultaneous ≤4% decrease in surface-negative CD3 [sCD3^−^] IELs) was strongly associated with active and potential forms in over 80% of the confirmed patients with CD, whereas the remaining patients with CD had partial lymphogram profiles (≥14% increase in TCRγδ or ≤4% decrease in sCD3^−^ IELs), with lower diagnostic certainty. None of these patients had a non-celiac lymphogram. Quantifying the TCRγδ versus sCD3^−^ imbalance as a ratio (≥5) is a discriminative index to discard or suspect CD at diagnosis. Conclusions: We have validated the IEL lymphogram’s diagnostic efficiency (79% sensitivity, 98% specificity), with an LR+ accuracy of 36.2. As expected, the increase in TCRγδ IELs is a reliable marker for celiac enteropathy, while changes in sCD3^−^ IEL levels throughout the dynamic CD process are useful biomarkers of mucosal lesions.

## 1. Introduction

Celiac disease (CD) is an immune-mediated enteropathy [1,2] that can affect genetically susceptible individuals [3,4] at all ages of life [5,6] and with an increasing prevalence in recent decades, at approximately 0.5–1.5% [7]. Gluten, a major source of protein nutrients worldwide, is the driver of the specific CD4^+^ T cell activation [8] that initiates the CD pathogenic process. Cytokine-mediated innate activation of the cytotoxic CD8^+^ T intraepithelial lymphocytes (IELs) [9] is necessary to induce the enterocyte damage that characterizes the atrophic CD lesion [10]. Although the wide spectrum of intestinal and extra-intestinal clinical manifestations is the consequence of malabsorptive enteropathy, it also stems from systemic autoimmune-like inflammation [3,11,12].

CD diagnosis in adults relies on the detection of CD-specific serology (IgA anti-transglutaminase antibodies [TG2] and anti-endomysial antibodies) [13,14,15] and increased infiltration of duodenal intraepithelial lymphocytes with variable degrees of villous atrophy [16], which still requires a confirmatory biopsy [17], despite ongoing debate in the field [18,19,20]. Both diagnostic pillars have relevant drawbacks that make CD diagnosis a challenge. Almost 2–15% of adult patients with CD with a compatible atrophic lesion can be primarily IgA-TG2 negative [21], or occasionally seronegative, as the result of associated immunodeficiencies, immunosuppressor treatments, and/or immune dysregulation [22]. Additionally, none of the histological changes that characterize CD enteropathy are pathognomonic [23,24], with many potential histopathological mimics [3] and, sometimes, with mild or patchy lesions which give rise to highly variable inter-observer interpretation [25]. Complicating this scenario, it is not unusual to find patients who have a reduced gluten intake without a definitive clinical diagnosis, with the consequent decrease in specific serological markers, favoring the healing of the mucosa.

The essential immune finding in active celiac enteropathy is the increase in IELs [26], a heterogeneous cell compartment which comprises the cytotoxic IEL effectors involved in enterocyte killing [27,28,29] and which is characterized by an increase in the T cell receptor (TCR)γδ IEL subset [30,31] and a decrease in the surface CD3-negative (sCD3^−^) IEL subset [32,33]. The flow cytometry assessment of this intraepithelial compartment, termed the “IEL lymphogram” [34,35,36], shows an almost pathognomonic “celiac lymphogram” profile, characterized by a long-lasting imbalance in the ratio of TCRγδ versus sCD3^−^ IEL subsets.

In the last decade, several groups have reported the utility and accuracy of the IEL lymphogram in the routine diagnosis of CD at any age, even in atypical forms or mucosal stages, according to the natural dynamics of the disease [37,38,39,40,41,42,43,44,45,46,47,48,49].

The aim of this study was to validate the diagnostic reliability of the lymphogram with the largest adult series to our knowledge, attempting to harmonize small discrepancies among laboratories in the interpretation of the obtained analytical parameters, adding new insights regarding their value as biomarkers of the dynamic process which occurs in celiac mucosa.

## 2. Materials and Methods

### 2.1. Patients

This was a retrospective study conducted at the Adult Gastroenterology Unit and the Department of Immunology of the Ramón y Cajal University Hospital (Madrid, Spain) between 2010 and 2020, as the result of routine clinical practice. As the inclusion criteria, only patients over 17 year of age with an available histological report and a clinical diagnosis were considered. The final diagnosis was made by a medical specialist in gastroenterology, based on the combination of clinical symptoms, specific CD serology, genetics, and duodenal mucosa histology, as recommended by several guidelines [3]. Duodenal IEL lymphogram analysis by flow cytometry was performed as part of the diagnostic protocol. Demographic data, specific CD serology, celiac genetics, duodenal histology, final clinical diagnosis, and TCRγδ/sCD3^−^ IEL percentages were registered in a private database. A total of 768 patients were included, in whom an upper endoscopy was indicated to discard CD. The patients were classified into the following groups (baseline characteristics are summarized in Table 1):

1. Control group (*n* = 411). All patients in this group were on a gluten-containing diet and lacked circulating IgA anti-TG2 antibodies. We have intentionally highlighted a Control Marsh stage I (MI) subgroup of 57 patients with duodenal lymphocytosis in whom CD was excluded (negative serology and/or non-compatible genetics and/or no clinical remission after gluten withdrawal). The remaining individuals included in this group (*n* = 356) showed no evidence of mucosal lesion (M0, Marsh stage 0).
2. Active CD seropositive (*n* = 203). The active CD group included only patients with a new diagnosis of CD on a gluten-containing diet. Serological tests were considered valid if performed between 1 year before and 1 year after the date of the endoscopy. In this group, almost all patients had positive IgA anti-TG2 serology, confirmed by anti-endomysial antibody. All patients presented villous atrophy (Marsh stage III a-c lesion).
3. Active CD seronegative (*n* = 14). A small group of patients with a new diagnosis of CD in a gluten-containing diet, lacked serum IgA anti-tTG2 antibodies. All patients presented villous atrophy (Marsh stage III a-c lesion).
4. Patients with CD in remission (gluten-free diet [GFD] group, *n* = 195). Patients in this group had been on a gluten-free diet (GFD) since their diagnosis (median 72 months, Q1–Q3 quartiles 36–156 months). Monitoring of diet compliance was performed by detecting the presence of specific CD serology. In general, a clinical visit was scheduled every 6 months until it became negative and annually thereafter. Only the specific CD serological tests performed between 3 months before and 3 months after the endoscopy were considered, to maintain a temporal agreement with the histological findings. A follow-up group (*n* = 70) was included, in which a second biopsy was required to demonstrate mucosal recovery or comorbidities unrelated to CD.
5. Patients with potential CD (*n* = 15). In this group, patients had positive specific serology without villous atrophy (Marsh 0-I). A permissive HLA-DQ2/DQ8 genotype was present when available (73%). Five asymptomatic patients were detected in family screening, seven had mild digestive symptoms, one had iron deficiency anemia, and two had an autoimmune disease. All initiated a GFD.

### 2.2. Small-Bowel Biopsy and Flow Cytometry Analysis

The biopsies for the histological analysis and one duodenal biopsy for the flow cytometry analyses were obtained during the same endoscopy. Mucosal morphology was classified according to the Marsh criteria, as modified by Oberhuber [50].

The biopsies for the flow cytometry analyses were collected in a saline solution if they were to be processed within 2 h or in RPMI cellular complete medium (see below) if processing was to be postponed for up to 12 h. Processing should not be delayed longer than 12 h. The biopsies included in this study were taken from the distal duodenum. Single-cell suspensions were prepared from the epithelial layer of the biopsy samples, employing a previously described protocol [51]. Briefly, IEL and epithelial cells were released from the mucosal specimens by incubation for 1 h under gentle agitation with 1 mM ethylenediaminetetraacetic acid (EDTA) and 1 mM dithiothreitol (DTT) (Sigma-Aldrich Corp., St. Louis, MO, USA) in RPMI 1640 medium (GibcoBRL Life technologies, Gaithersburg, MD, USA) supplemented with 10% fetal calf serum. The released cell suspension was collected by centrifugation, washed, and stained with fluorochrome-conjugated monoclonal antibodies (mAbs). The cells were surface-stained for 30 min on ice with mAbs against CD45 (APC conjugated, clon HI30), CD3 (PerCP conjugated, clon SK7), TCRγδ (PE conjugated, clon 11F2), and CD103 (FITC conjugated, clon Ber-ACT8), all from BD-Pharmingen, to quantify the percentage of IELs relative to the epithelial cells and the percentage of CD3^+^ TcRγδ and sCD3^−^ CD103^+^ IEL subsets relative to the total IELs. IEL marker expression was analyzed by 4-color flow cytometry in a FACSCanto (BD Biosciences, Franklin Lakes, NJ, USA), and the data were processed with the DIVA software version 8.0.2 (BD Biosciences). Total IELs were selected from the entire cell population of epithelial cells based on their low 90° light scattering and CD45 expression.

All biopsy specimens were obtained for diagnostic purposes after informed written consent had been collected and in accordance with the ethical guidelines of our institution.

### 2.3. Statistics

Absolute and relative frequencies were employed to describe categorical variables. Median and interquartile range (Q1–Q3) were used to describe continuous variables.

For the flow cytometry analysis, the following variables were considered: (a) % of TCRγδ relative to the total IELs; (b) % of sCD3^−^ relative to total IELs; and (c) ratio of TCRγδ versus sCD3^−^. Differences in these variables between the groups were tested with a non-parametric analysis of variance (Kruskal–Wallis), followed by Dunn’s multiple comparison test. Differences between matched-pair data were tested with the Wilcoxon signed-rank test.

Receiving operator curve (ROC) analyses were employed to define the best cut-offs for the score, optimizing sensitivity and specificity to calculate the performance of the above-defined variables in CD diagnosis. Sensitivity, specificity, and positive and negative likelihood ratios (LR+ and LR−) and their 95% confidence intervals (CIs) were calculated. We took LR+ >10 and LR− <0.1 as convincing diagnostic evidence. Disease probability for each lymphogram profile was calculated as the percentage of patients with CD who presented a defined lymphogram profile relative to the total number of individuals who presented the same profile. Statistical analyses were performed with GraphPad Prism 5.0 (GraphPad Software, Inc., San Diego, CA, USA). A *p*-value < 0.05 was taken as statistically significant. The illustrations in this paper were created with Prism GraphPad Software version 8.0.2 (La Jolla, CA, USA).

## 3. Results

### 3.1. TCRγδ and sCD3^−^ Intraepithelial Lymphocyte Subsets: Distribution among Celiac Disease Groups and Controls

**Figure 1:**
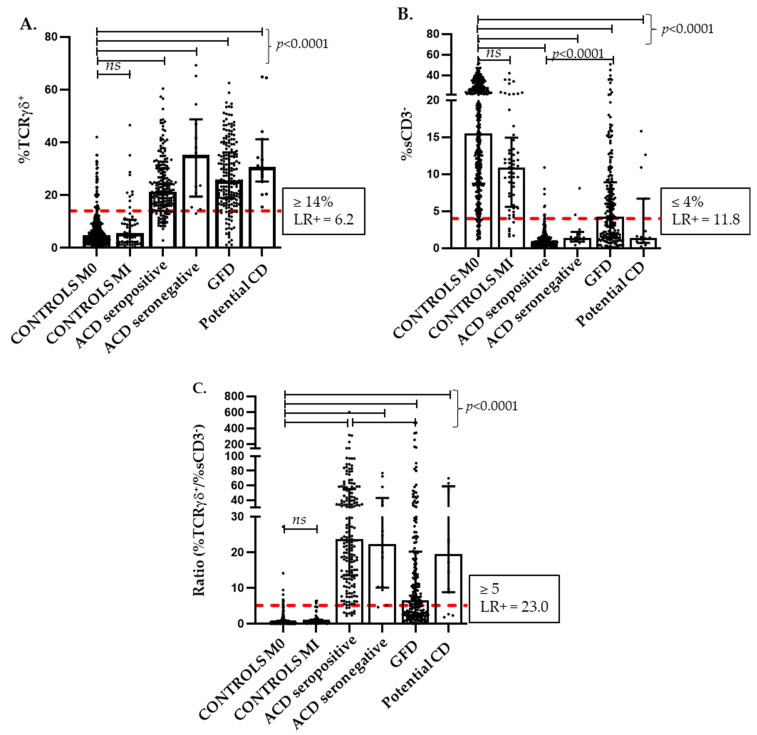
Distribution of the TcRγδ (**A**) and sCD3^−^ (**B**) IEL subset percentages and of the calculated ratio between % TcRγδ^+^/% sCD3^−^ (**C**) in the different forms of celiac disease and the controls. % TcRγδ^+^ and % sCD3^−^ IEL densities are expressed as a percentage of the total intraepithelial lymphocytes. Discriminative cut-offs are indicated as dashed lines on the y-axes and are calculated by ROC curve analysis. Median and quartiles are shown over the individual pointed data. ACD, active celiac disease; CD, celiac disease; GFD, gluten-free diet; M0, Marsh type 0; MI, Marsh type I; and ROC, receiver-operating characteristic curve. *p* values were calculated using the non-parametric ANOVA (Kruskal–Wallis) test, *ns*: not statistically significant.

Figure 1A confirms the significantly higher percentages of TCRγδ IELs in all the defined CD groups compared with the controls (*p* < 0.0001), which reinforces its value as a CD biomarker throughout the dynamic process of the disease. No significant differences in the TCRγδ IEL counts were observed between the various forms of CD, not even when patients with active CD were grouped by age (*n* = 171, aged 17–49 years; *n* = 36, aged 50–69 years; and *n* = 10, aged 70–99 years), with TCRγδ median percentages (Q1–Q3) of 22.2% (16.2–32.7), 22.3% (16.0–28.8), and 20.3% (14.4–24.7), respectively; thus, we have applied a unique discriminative cut-off point in the analytical statistics.

In parallel, Figure 1B illustrates how sCD3^−^ IEL densities significantly drop in either CD group (*p* < 0.0001) when compared with the control group (median 15.5%; Q1–Q3 8.7–25.7), with a higher score in active CD forms (median 0.8%; Q1–Q3 0.5–1.4) but with a significant rising trend (*p* < 0.0001) in the less-atrophic CD forms after gluten withdrawal (median 4.3%; Q1–Q3 1.5–8.9), conferring to this parameter a significant value as a mucosal integrity sensor.

Table 2 details some descriptive statistics of both cytometric parameters either as independent variables or as a combined ratio between TCRγδ and CD3^−^ IEL. This highlights the wide individual variations in TCRγδ and sCD3^−^ IELs in each group, the overlap among groups, and the dynamic IEL subset changes during the celiac process.

### 3.2. TCRγδ and sCD3^−^ Intraepithelial Lymphocyte Subsets: Establishing Cut-Offs and Defining Lymphogram Profiles

The optimal cut-off values for the TCRγδ and sCD3^−^ IEL subsets were obtained from the ROC curves. A cut-off of TCRγδ IEL density ≥ 14% and of sCD3^−^ IEL density ≤ 4% optimized the sensitivity and specificity to calculate the best performance of these independent variables to predict CD at diagnosis (Table 3). Notice that the isolated evaluation of sCD3^−^ ≤ 4% yields a higher sensitivity and specificity than the isolated evaluation of TCRγδ ≥ 14% for the detection of active CD forms. More relevant, however, is the combination of both parameters, TCRγδ and sCD3^−^, which are superior in the detection of CD than each separately, as can be observed when we apply the ratio of TCRγδ/sCD3^−^ as an index of the long-standing imbalance that characterizes the celiac lymphogram. A cut-off of ≥5 for this ratio has the best discriminative LR (LR+ 24.38 and LR− 0.05) (Table 3) calculated from the ROC curve analysis. Figure 1C shows the distribution and significances of this ratio among the different groups.

**Figure 2:**
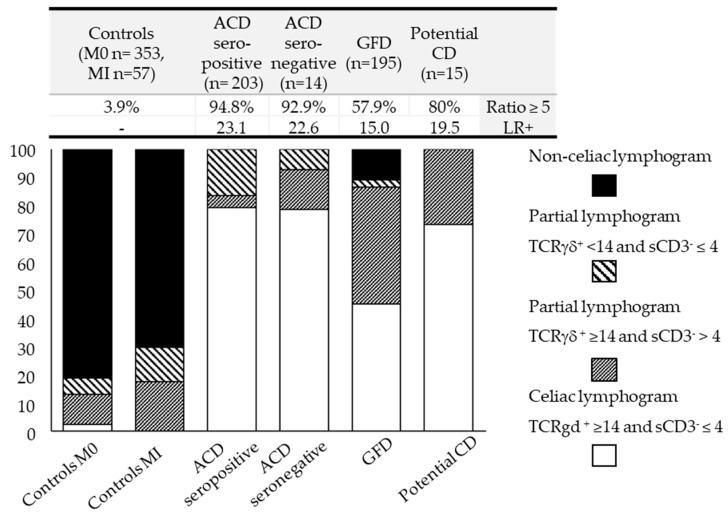
Distribution of the four different lymphogram profiles in the different forms of celiac disease and the controls. Lymphogram profiles combine TcRγδ and sCD3^−^ IEL variables according to their compliance with the established cut-offs. The data table at the top of the figure shows the percentage of patients in each group that meet the TCRγδ/sCD3^−^ ≥ 5 ratio requirement and the calculated diagnostic accuracy (LR+) for each one. ACD, active celiac disease; CD, celiac disease; GFD, gluten-free diet; and LR+, positive likelihood ratio.

To go deeper into the analysis of the IEL lymphogram throughout the dynamic process of CD, we have defined four distinct lymphogram profiles according to compliance with the above-established cut-offs when combining both variables: a complete celiac lymphogram (TCRγδ ≥ 14 and sCD3^−^ ≤ 4%), two partial lymphograms (one supported by an increase in TCRγδ ≥ 14% with sCD3^−^ > 4% and the other supported by decreased sCD3^−^ density ≤ 4% with TCRγδ < 14%), and a non-celiac lymphogram (TCRγδ < 14% and sCD3^−^ > 4%). Figure 2 shows the lymphogram profile results for each CD group and the controls. Interestingly, the upper data table in Figure 2 shows the correlation between the lymphogram performance in each group and the percentage of patients in each group meeting the TCRγδ/sCD3^−^ ≥ 5 ratio requirement, supporting that this index is associated with convincing diagnostic evidence of CD in each group (LR+).

A majority (81%) of the Marsh 0 control group had a non-celiac lymphogram, 16% had partial lymphograms, mainly based on an isolated increase in TCRγδ ≥ 14%, and 2.5% showed a complete celiac lymphogram (mostly women with dyspepsia and/or anemia in the context of autoimmune pathology, mainly gastritis and thyroiditis). The two individuals with non-celiac villous atrophy included in this group (Table 1) had non-celiac IEL distributions and a TCRγδ/sCD3^−^ ratio of <5. In the differentiated Marsh I control group (*n* = 57), 70.1% (*n* = 40) had non-celiac lymphograms. Of the remaining 17 individuals who had partial lymphograms, 14 had a TCRγδ/sCD3^−^ ratio of <5. Thus, only in three patients was the lymphogram inconclusive: two had parasites in their stool and one was lactose intolerant, this ratio emerging as an efficient marker to discard active CD in atypical patients.

The largest fraction (≈80%) of patients with active CD fit the complete celiac lymphogram profile, with a 95% disease probability, whereas the remaining patients (≈20%) presented partial lymphograms based on an isolated increase in TCRγδ ≥ 14% (4.6%) or an isolated decrease in sCD3^−^ density ≤ 4% (15.7%), both with a much lower disease probability (17% and 55% disease probability, respectively). Almost 95% of the patients in this group had a TCRγδ/sCD3^−^ ratio of ≥5, supporting the good performance of this index when interpreting partial lymphograms.

Notice that potential forms (although the low number of patients included precluded us from drawing robust conclusions) were also efficiently detected, with 70% of the patients presenting a complete celiac lymphogram (11 of the 15), and the remaining 4 patients showing partial lymphograms, mainly at the expense of isolated increases in TCRγδ ≥14%. The TCRγδ/sCD3^−^ ratio of ≥5 showed a behavior parallel to the lymphogram profile.

None of the patients with CD at diagnosis (*n* = 232) (active or potential) had a non-celiac lymphogram profile, which reinforces the high negative predictive value of a non-celiac lymphogram profile or a TCRγδ/sCD3^−^ ratio of ≤5 in discarding CD.

A particular circumstance was present in the GFD group, with only 45% presenting a complete celiac lymphogram, 45% with partial lymphograms mainly at the expense of isolated TCRγδ ≥ 14% increases, and 10% presenting non-celiac lymphograms, with the consequent loss of diagnostic accuracy, also reflected in the drop in the percentage of GFD patients with a TCRγδ/sCD3^−^ ratio of ≥5 from 94.8% in active CD to 57.9% in GFD CD.

The diagnostic efficiency (Table 4) of the complete celiac lymphogram when both variables fit the cut-off values (TCRγδ ≥ 14 and sCD3^−^ ≤ 4%) was 79% sensitivity and 98% specificity (with a diagnostic accuracy of LR+ 36.2; 95% CI 18.9–69.4), improving the sensitivity to 100% but lowering the specificity to 80%, when partial lymphograms were also considered.

### 3.3. TCRγδ and sCD3^−^ Intraepithelial Lymphocyte Subsets: Biomarkers of the Ongoing Immunological Celiac Disease Process

**Figure 3:**
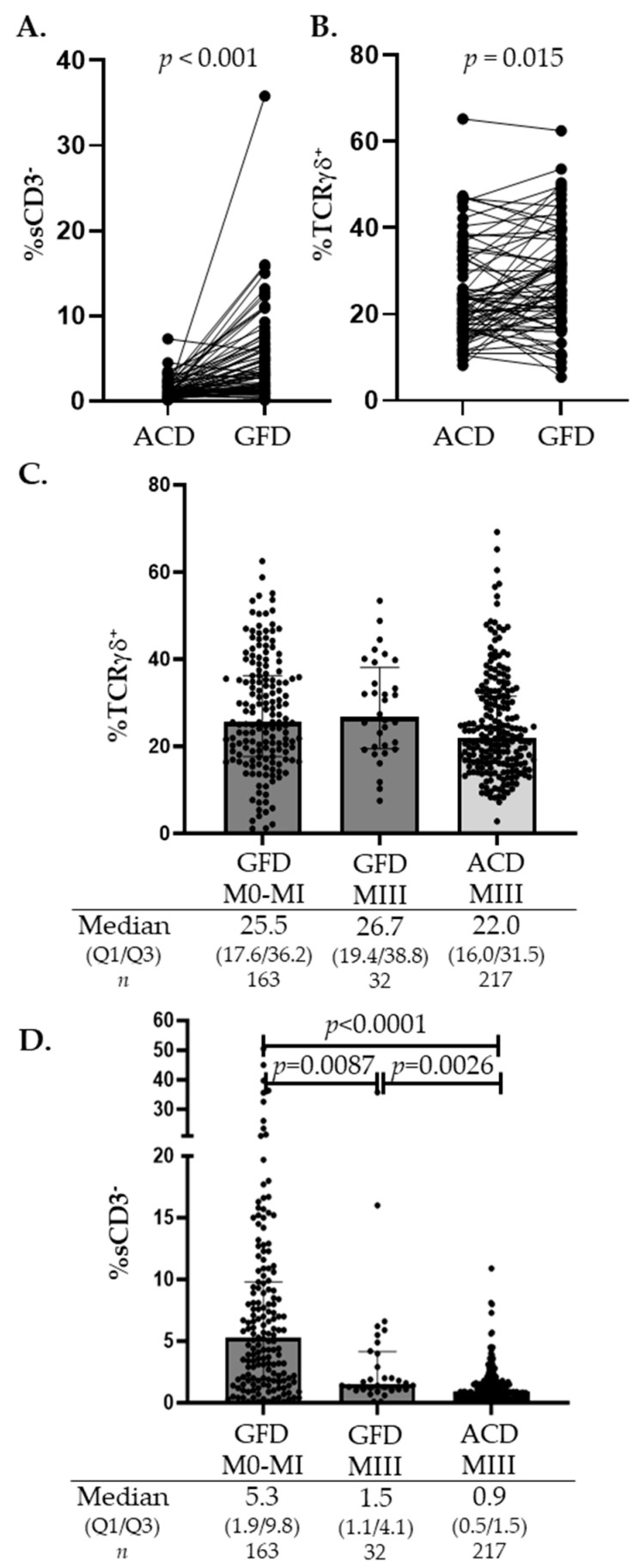
Distribution of the TcRγδ and sCD3^−^ percentages in patient on a GFD. (**A**,**B**) represent a follow-up series (*n* = 70) (ACD → GFD) comparing the percentages of sCD3^−^ IELs and TcRγδ IELs in active CD patients at diagnosis with follow-up after gluten withdrawal. Matched-pair data were tested with the Wilcoxon signed-rank test. (**C**,**D**) represent the distribution of the TcRγδ and sCD3^−^ subsets in the full GFD cohort (*n* = 195), grouped according to the biopsy histological findings and compared with the atrophic ACD group. *p* values were calculated using the non-parametric ANOVA (Kruskal–Wallis) test. ACD, active celiac disease; CD, celiac disease; GFD, gluten-free diet; M0, Marsh type 0; MI, Marsh type I; MIII, Marsh type III; *n*, number; and Q1/Q3, quartile 1/quartile 3.

Patients on a GFD represent a unique group for monitoring IEL changes induced by gluten. Our cohort included 70 patients who underwent a follow-up biopsy after gluten withdrawal from their diet. The median time between the first diagnostic biopsy and the follow-up biopsy was 35.3 months (Q1–Q3, 25.1–48.5). Figure 3A illustrates the significant increasing trend in sCD3^−^ IEL densities after initiating the GFD, from a median of 0.9% before the GFD (Q1–Q3, 0.4–1.5) to a median of 3.1% after the GFD (Q1–Q3, 1.8–6.6), whereas changes in the TCRγδ IEL percentages were widely overlapping (Figure 3B). These trends were reinforced when analyzing the global GFD group (*n* = 195). Figure 3C shows the persistent elevation in TCRγδ, with no highly significant differences when these patients were grouped by the level of mucosal lesion, or even when compared with the TCRγδ densities in the active CD group with Marsh III lesions. In contrast, although always decreased if compared with a healthy mucosa, differences in sCD3^−^ IEL densities were significant between the GFD patients with villous atrophy (Marsh III) and those GFD patients with healing mucosa (Marsh 0-I), even when compared with sCD3^−^ IEL densities in the atrophic active CD group (Figure 3D). There is a clear rising trend in sCD3^−^ IEL cells after gluten withdrawal, probably related to the ceasing of gluten-induced inflammation, which culminates in mucosal recovery.

## 4. Discussion

Each patient with CD develops a unique immunological response elicited by gluten that is conditioned by the genetic background of each individual and is presumably modulated by environmental factors [52,53,54]. The resultant inflammatory enteropathy is a dynamic process that comprises a gluten-specific CD4 T cell response that occurs at the local lymph nodes and lamina propria, which culminates with the innate cytotoxic destruction of the enterocytes [28]. Both levels of the immune response must be finely regulated and coupled, giving rise to the wide spectrum of clinical CD forms; however, the precise mechanisms are not yet completely understood.

One of the essential findings of this active process is the increase in IELs [26,27]; flow cytometry analysis has facilitated the monitoring of IEL subsets in various outcomes throughout the natural history of the disease. More than a decade ago, our group coined the term “IEL lymphogram” to describe the near-pathognomonic imbalance in the ratio of TCRγδ to sCD3^−^ IELs in a pediatric CD cohort [33,34,35]. Since then, several groups have proven the diagnostic utility of the lymphogram either in pediatric or adult series [37,38,39,40,41,42,43,44,45,46,47,48,49]. In the present study, we add further evidence to the reliability of this technique by analyzing a large adult CD cohort that supports previous findings, driving conclusions and recommendations on lymphogram interpretation.

On a first descriptive analysis of the distribution of the TCRγδ and sCD3^−^ IEL cytometric variables among our defined groups (Figure 1), we found that the TCRγδ subset density increased ≥14% in 86% of our global celiac cohort, confirming this parameter as the characteristic immune marker of the celiac process throughout its various stages and forms, as widely documented [30,31,55]. However, it is not a pathogenic finding of CD, as has previously been reported in other situations, such as food intolerances, giardiasis, cryptosporidiosis, Sjögren’s syndrome, or IgA deficiency [56,57,58], and which was present in 13% of the individuals included in our control group, yielding a CD diagnostic accuracy of LR+ 6.2. In contrast, the drastic drop in sCD3^−^ IELs ≤ 4% resulted in a more efficient predictor of active CD, with LR+ 11.8. However, it was the combination of both parameters, expressed as the ratio of TCRγδ/sCD3^−^, that gave rise to the best CD predictor index (Table 3, Figure 1C) with LR+ 23.

When analyzing the distribution of the various IEL lymphogram profiles in the CD groups at diagnosis, we verified that almost 80% of the patients had a complete celiac lymphogram (176 of 223), with a 95% probability of the disease. The remaining 20% had partial lymphograms, associated with a lower probability of the disease. In this context, a partial lymphogram due to the isolated ≤4% decrease in sCD3^−^ IELs showed a higher disease probability than when the partial lymphogram was due to the isolated increase of ≥14% in TCRγδ (57% versus 23%, respectively), as previously discussed. We validated the celiac lymphogram as a specific and sensitive detector of active CD, as already supported by previously cited studies; this is especially relevant in patients with seronegative CD [40,43,59] and potential CD [49,60] and in differential diagnoses with other atrophic enteropathies [3].

When gluten is withdrawn from the diet, the mucosa initiates a healing process in which TCRγδ and sCD3^−^ subsets experience dynamic changes that can be monitored with a lymphogram. In our GFD group, 45% of the patients displayed a complete celiac lymphogram and 41% presented partial lymphograms, mainly based on the isolated ≥14% increase in TCRγδ density as a hallmark of the celiac condition [37,54,61,62], accompanied by a rise in sCD3^−^ IEL counts, as a marker of mucosal recovery and GFD compliance [35,37]. There was a significant increase in the sCD3^−^ IEL counts in the GFD patients with an optimal mucosal recovery compared with the GFD patients, in which mucosal lesions persisted, and between these patients compared to patients with active CD (Figure 3D). This sCD3^−^ subset is highly represented in healthy mucosa and almost disappears under the celiac inflammatory cascade responsible for villous atrophy, becoming a sensitive marker of mucosal lesions. The function of these sCD3^−^ IELs is poorly defined [29,63], comprising a diversity of innate lymphoid cells and precursor lymphoid cells [9,29,64]. Still, in this GFD scenario, 11% of our treated patients had a non-celiac lymphogram, which could indicate a natural tendency to resolve the TCRγδ versus sCD3^−^ imbalance after gluten withdrawal. Gluten intake might be a pivotal modulator of TCRγδ functionality and/or number [65], and the persistence of small traces of it in a diet could be responsible for the long-lasting presence of these lymphocytes in celiac mucosa, although their pathogenic role in CD remains unclear. Published data describe changes in the TCRγδ IEL receptor repertoire and functional reprogramming toward regulatory or inflammatory pathways induced by celiac inflammation [66,67,68,69].

Potential CD is a celiac condition whose natural progression is not well understood, in which patients develop a gluten-specific CD4^+^ T cell response, with serum-positive celiac antibodies, but in the absence of epithelial cytotoxic damage, which appears to be somehow uncoupled in the sequential celiac process [70]. Our cohort, not large enough (*n* = 15) to obtain robust significance, still offered interesting observations: all of them had an increase of ≥14% in TCRγδ IELs, a valuable marker of CD, accompanied, in four of them, by the concomitant increase in sCD3^−^ counts, a marker of a lower risk of progression to villous atrophy, which validated the utility of the lymphogram in the detection and monitoring of potential CD, in line with previous studies [37,44]. Coincident results were obtained when using the ratio TCRγδ/sCD3^−^ of ≥5 as a discriminative index, with an LR+ of 19.5.

This is not a prospective study but rather the result of the routine diagnosis and follow-up of patients with CD in our clinical practice, which may be a limitation, mainly in the follow-up group analysis, where we could not assure compliance with the diet. As a strength of the procedure, we highlight the transferability of the results between sites, as the diagnostic efficiency of the lymphogram obtained in this study is equivalent to those referred by other groups already included in our discussion.

The IEL lymphogram assay was initially set up by our group in the framework of several research projects and was subsequently included in the routine protocol of CD diagnosis in our hospital, more than two decades ago [35]. Since 2018, it has been included in a national guide for early CD diagnosis [71]. Based on our experience, we propose the following recommendations:

- Knowing whether a patient is ingesting gluten is critical when interpreting partial lymphogram profiles in the initial diagnosis of CD: ↑TCRγδ ≥ 14 and ↑sCD3^−^ > 4% in a patient eating gluten practically excludes an active CD form or introduces the option of a potential CD form, whereas, if the patient is on a GFD, it indicates good follow-up.
- Chosen cut-offs are arbitrary and depend on the priorities of the clinicians, favoring specificity or sensitivity, so it is important to be aware that the higher the specificity, the better the discriminatory power.
- Quantifying the TCRγδ/sCD3^−^ imbalance ratio is a good discriminative index to discard or suspect an active CD form.
- The lymphogram is a simple, rapid, and accurate technique, but it requires expertise in mucosal immunology to interpret and further analyze the profound phenotypic or numerical changes in the dynamic IEL compartment.

## 5. Conclusions

We validated the following points, in line with the numerous studies cited in this work:

- The celiac lymphogram is a highly specific imprint of the subjacent immunopathogenic process, guided by gluten intake.
- An increase in TCRγδ IELs is the pathological hallmark of CD enteropathy in the majority of CD forms.
- The sCD3^−^ IEL subset is a sensor of celiac mucosal integrity, almost disappearing in active CD forms but with an increasing tendency in healing mucosa.
- A complete celiac lymphogram has a high diagnostic accuracy (LR+ 36.2).
- A non-celiac lymphogram practically excludes active CD.
- Once a diagnostic or follow-up biopsy is clinically indicated, the lymphogram confers specificity to the histological findings and increases the efficiency of the whole diagnostic process.

## Figures and Tables

**Table 1:** Baseline characteristics of all groups.

	Controls	ACD Seropositive	ACD Seronegative	GFD	Potential CD
**TOTAL**	411	203	14	195	15
**Sex: *n* [%]**	M: 121 [30] F: 289 [70]	M: 54 [27] F: 149 [73]	M: 4 [29] F: 10 [71]	M: 56 [29] F: 139 [71]	M: 4 [36] F: 11 [64]
**Age (years)**					
**Median**	41	36	44	39	35
**(min/max)** **Q1/Q3**	(17/88) 31/50	(17/83) 26/47	(20/90) 26/66	(17/85) 27/52	(19/68) 31/42
**Villous atrophy *n* [%]**					
**Marsh 0**	353 [85.9]	0	0	129 [66.2]	9 [60]
**Marsh I**	57 [13.9]	0	0	34 [17.4]	3 [20]
**Marsh II**	1 [0.2]	0	0	3 [1.5]	3 [20]
**Marsh III A, B, C**	0	203 [100]	14 [100]	29 [14.9]	0
**Serology *n* [%]**					
**Positive**	0	194 [95.5]	0 [0]	9 [4.6]	15 [100]
**Negative**	412 [100]	0	14 [100]	112 [57.4]	0
**Not available**	0	9 [4.5]	0 [0]	74 [38.0]	0
**HLA allele *n* [%]**					
**DQ2 or DQ8**	92 [22.3]	114 [56.1]	12 [85.7]	115 [59.0]	11 [73.3]
**Nor DQ2 or DQ8**	41 [10.3]	2 [1.0]	0 [	1 [0.5]	0
**Not available**	277 [67.4]	87 [42.9]	2 [14.3]	79 [40.5]	4 [26.7]

ACD, active celiac disease; CD, celiac disease; GFD, gluten-free diet; F, female; M, male.

**Table 2:** Distribution of TcRγδ and sCD3^−^ IEL subsets in CD groups and controls, as independent cytometric variables and as the calculated ratio between TcRγδ and sCD3^−^.

	Controls M0	Controls MI	ACD Seropositive	ACD Seronegative	GFD	Potential CD
TOTAL	353	57	194	14	195	15
% TCRγδ^+^						
median	4.8	5.5	21.35	35.25	25.5	30.6
Q1/Q3	2.6/9.2	2.1/10.7	16.1/30.2	19.4/48.8	18.7/36.2	25.1/41.2
min/max	0.2/42.0	0.4/46.5	2.8/60.4	13.0/69.2	1.1/62.5	15.5/64.8
% sCD3^−^						
median	15.5	10.9	0.8	1.4	4.3	1.4
Q1/Q3	8.7/25.7	5.6/15.0	0.5/1.4	0.9/2.2	1.5/8.9	0.7/6.7
min/max	1.2/72.8	1.6/42.0	0.1/10.8	0.4/8.1	0.1/50.5	0.2/15.8
median	0.3	0.6	23.8	22.3	6.5	19.4
Q1/Q3	0.1/0.9	0.2/1.2	13.5/55.1	10.0/43.0	2.5/20.2	8.8/58.9
min/max	0.004/27.2	0.03/6.4	2.2/604	4.6/76.9	0.03/470	1.8/135.0

% TcRγδ^+^ and % sCD3^−^ IEL densities are expressed as a percentage of the total intraepithelial lymphocytes. ACD, active celiac disease; CD, celiac disease; GFD, gluten-free diet; M0, Marsh type 0; and MI, Marsh type I.

**Table 3:** Statistical performance of duodenal TcRγδ and sCD3^−^ IEL subsets in CD prediction, either as independent cytometric variables or as the combined ratio of TcRγδ versus sCD3^−^.

	Cut-Off	AUC	Sensitivity (95% CI)	Specificity (95% CI)	LR+	LR−
**% TCRγδ** **^+^**	≥14%	0.917	84.33 (78.80–88.90)	86.37 (82.67–89.54)	6.19	0.18
**% sCD3** **^−^**	≤4%	0.984	94.91 (91.07–97.43)	91.97 (88.91–94.41)	11.82	0.06
**ratio** **TCRγδ** **^+^** **/sCD3** **^−^**	≥5	0.992	94.91 (91.07–97.43)	96.11 (93.75–97.76)	24.38	0.05

Receiver-operating characteristic curve (ROC) analysis was used to define the best cut-offs points. AUC, area under the curve; CI, confidence interval; and LR, likelihood ratio.

**Table 4:** Diagnostic accuracy of IEL lymphogram profiles.

	Sensitivity (95% CI)	Specificity (95% CI)	PPV (95% CI)	NPV (95% CI)
**^a^ Complete celiac lymphogram**	0.79 (0.74–0.85)	0.98 (0.96–0.99)	0.94 (0.91–0.98)	0.91 (0.88–0.94)
**^b^ Complete celiac + partial lymphograms**	1.00 (0.98–1.00)	0.80 (0.76–0.83)	0.70 (0.64–0.75)	1.00 (0.99–1.00)

^a^ Both variables, TcRγδ IELs and sCD3^−^ IELs fit into the cut-off values that define a complete celiac lymphogram (≥14% increase in TCRγδ and simultaneous decrease of ≤4% in sCD3^−^ IELs). ^b^ When partial lymphograms are also considered (isolated ≥14% increase in TCRγδ or isolated ≤4% decrease in sCD3^−^ IELs). CI, confidence interval; NPV, negative predictive value; PPV, positive predictive value; LR, likelihood ratio.

## Data Availability

The private datasets generated and/or analyzed during the current study are available from the corresponding author upon reasonable request and only in the case of a research project approved by an Ethics Committee.
